# Impact of Lignin Substitution on the Formation and Physicochemical Properties of Resorcinol–Furfural-Based Carbon Aerogels in Deep Eutectic Solvent

**DOI:** 10.3390/polym18172122

**Published:** 2026-08-31

**Authors:** Rui Lou, Anqi Xu, Kelun Feng, Kewei Zhang, Yueyue Guo, Long He

**Affiliations:** College of Mechanical and Electronic Engineering, Shaanxi University of Science and Technology, Xi’an 710021, China

**Keywords:** lignin, carbon aerogels, deep eutectic solvent (DES), furfural, electrochemical performance

## Abstract

Aligned with green chemistry principles and the pursuit of sustainable synthesis, we developed a lignin–resorcinol–furfural (LRF) gel system within a deep eutectic solvent (DES), in which lignin partially replaces resorcinol and furfural serves as a bio-based alternative to formaldehyde. We further examined the correlation between the lignin substitution ratio (65–90%) and the gelation kinetics, microstructure, and electrochemical performance of the resulting carbon aerogels (LRFC). The findings reveal that the lignin substitution ratio exerts a critical influence on both the crosslinking uniformity and the overall network architecture of the resulting LRFC. The results demonstrated that LRFC65, with a lignin substitution ratio of 65%, exhibited the optimal electrochemical performance: a specific capacitance of 117.2 F g^−1^ at a current density of 0.2 A g^−1^, along with lower charge transfer and diffusion resistance. This work offers promising prospects for high-value bio-based furfural and lignin valorization.

## 1. Introduction

As one of the emerging electrochemical energy storage devices, supercapacitors (SCs) have demonstrated considerable promise in applications ranging from new-energy vehicles and stationary energy storage systems to portable electronic devices. This is due to their high power density, rapid charge–discharge capability, and exceptional cycling stability. Notably, the electrochemical performance of SCs is critically influenced by the structure and physicochemical properties of their electrode materials [1]. In this context, carbon aerogels (CAs) have attracted extensive research interest as next-generation SC electrodes, owing to their three-dimensional (3D) porous network architecture, high specific surface area, and favorable electrical conductivity [2,3,4,5].

Lignin, the most abundant naturally occurring aromatic biopolymer, contains numerous functional groups such as phenolic hydroxyl, carbonyl, and methoxy groups within its molecular structure. These features provide an intrinsic advantage for its conversion into high-performance bio-based carbon materials [6,7,8,9]. Consequently, lignin is increasingly replacing petroleum-derived phenolic compounds, such as phenol and resorcinol, in phenolic condensation reactions [10]. Due to the renewability and low cost of its precursor, lignin-based CAs hold significant promise as sustainable electrode materials for SCs, offering both developmental potential and environmental compatibility [11,12]. However, the predominant synthesis of lignin-based CAs relies heavily on formaldehyde as a crosslinking agent, which not only raises production costs but also presents considerable environmental and health concerns. In accordance with green chemistry principles, recent research has increasingly focused on substituting formaldehyde with bio-derived furfural in various applications, including bio-oil upgrading and phenolic resin synthesis [13]. Notably, Tian et al. [14] synthesized resorcinol–furfural resin-derived porous carbon in KOH solution using furfural as the crosslinker, and the resulting carbon exhibited a specific capacitance of 291 F g^−1^ at 0.1 A g^−1^ in SC applications. Tao et al. [15] developed a modified phenolic resin using lignin and furfural, achieving partial replacement of petroleum-based phenol and formaldehyde in wood adhesives. These findings underscore the feasibility of industrial-scale adoption of low-formaldehyde products.

Complementing this approach, deep eutectic solvents (DESs) have emerged as highly effective green reaction media [16]. They not only facilitate the efficient dissolution of lignin and reduce its steric hindrance but also promote thorough copolymerization between lignin and furfural, thereby enhancing lignin’s intrinsic reactivity without requiring additional chemical pretreatment [17,18]. For instance, Cao et al. [19] successfully prepared lignin–resorcinol–formaldehyde (LRF)-derived CAs in a DES cosolvent system using DES-fractionated lignin and formaldehyde as precursors. The resulting CAs featured a 3D interlinked framework with bead-like chains and exhibited a specific capacitance of 284 F g^−1^ at 0.5 A g^−1^.

In this study, we report, for the first time, an integrated green and sustainable system that combines lignin, furfural, and DES. In this system, DES-fractionated lignin serves as the renewable precursor, while furfural acts as a bio-based alternative to formaldehyde for constructing the LRF gels. The effects of various lignin substitution ratios on the microstructure and electrochemical performance of the resulting LRF-based CAs are systematically investigated. Consequently, this work holds promising industrial potential for the high-value valorization of lignin and furfural biomass.

## 2. Materials and Methods

### 2.1. Materials

The lignin feedstock was isolated from pine wood (Asia Symbol, Rizhao, China) using a choline chloride/lactic acid (ChCl-LA) DES with a molar ratio of 1:5. The reaction conditions: temperature of 130 °C, time of 24 h, a solid-to-liquid mass ratio of 1:10. The previously established procedures and the chemical structure of extracted lignin are detailed in our earlier work [19].

### 2.2. Preparation of LRF

LRF gels with lignin substitution ratios of 65%, 75%, 85%, and 90% were prepared by dissolving lignin and resorcinol (total mass of 400 mg) together with 400 mg of furfural in 8 mL of ChCl-LA DES at a molar ratio of 1:5. The resulting mixture was then incubated at 90 °C for 6 h to gelation. Specifically, the lignin substitution ratio is defined as the mass percentage of lignin replacing resorcinol in the precursor formulation, while this mass-based substitution cannot be directly equated to equivalent substitution of phenolic reactive sites. The gelation time was determined by the vial inversion method [19]. After incubation, the vial was inverted and kept static for 30 s. The gelation point was defined as the time at which the sol ceased to flow upon vial inversion. All measurements were performed in triplicate, and the average gelation time was recorded.

After completion of the reaction, the wet gels were immersed in a *n*-butanol/water mixture for the removal of residual DES, and subsequently subjected to freeze-drying at −70 °C under 0.1 mbar to yield the final products. Accordingly, the obtained gels were designated as LRF65, LRF75, LRF85, and LRF90, corresponding to the respective lignin substitution ratio.

### 2.3. Fabrication of LRFC

The LRF samples were placed in a tube furnace and pyrolyzed under nitrogen atmosphere with a flow of 200 mL min^−1^. The furnace was heated from room temperature to 500 °C at a heating rate of 5 °C min^−1^ for 2 h, then further increased to 800 °C at the same rate for 3 h, followed by natural cooling to room temperature. The resulting CA products (LRFC) were labeled as LRFC65, LRFC75, LRFC85, and LRFC90.

### 2.4. Characterizations

Fourier transform infrared (FTIR) spectroscopy was performed using a Bruker Vertex 70 spectrometer (Bruker Optics GmbH & Co. KG, Ettlingen, Germany), and spectra were collected by the KBr pellet method over the range of 400–4000 cm^−1^ at a resolution of 4 cm^−1^ and a scanning speed of 32 scans s^−1^. The microscopic morphology was observed by field-emission scanning electron microscopy (SEM, FEI Verios 460, Thermo Fisher Scientific Inc., Hillsboro, OR, USA) after gold sputtering. X-ray diffraction (XRD) patterns were acquired on a SmartLab 9 kW X-ray diffractometer (Rigaku Corporation, Tokyo, Japan) using monochromatic Cu Kα radiation (λ = 0.154056 nm) as the light source, over the 2*θ* range of 10–80° at a scanning speed of 5° min^−1^. Raman spectra were measured by a DXRxi laser micro-Raman imaging spectrometer (Thermo Fisher Scientific Inc., Waltham, MA, USA) to characterize the lattice structural features of the carbon samples. The specific surface area and porosity were detected on an automated gas sorption analyzer (ASAP 2460 sn:506, Micromeritics Instrument Corporation, Norcross, GA, USA) with a flow of nitrogen using the Brunauer–Emmett–Teller (BET) method.

### 2.5. Electrochemical Measurements

The working electrodes were fabricated by mixing the active material (LRFC), acetylene black, and polyvinylidene fluoride (PVDF) in a mass ratio of 8:1:1 in N-methyl-2-pyrrolidone (NMP) to form a homogeneous slurry. The slurry was then coated onto nickel foam current collector (1 × 1 cm^2^) and dried at 80 °C for 12 h under vacuum. The mass loading of the active material on each working electrode was 2.0 ± 0.2 mg cm^−2^.

All electrochemical tests were performed in a three-electrode system using 6 M KOH aqueous solution as the electrolyte, with a platinum wire as the counter electrode and a Hg/HgO electrode as the reference electrode. The cyclic voltammetry (CV), galvanostatic charge–discharge (GCD), and electrochemical impedance spectroscopy (EIS) were conducted to assess the capacitive properties using a DH-7000d electrochemical workstation (Jiangsu Donghua Analytical Instrument Co., Ltd., Taizhou, China). CV curves were obtained within a potential window of −1 to 0 V at a scan rate of 5–100 mV s^−1^, along with GCD curves at the current density of 0.2–5 A g^−1^ and EIS at the open-circuit potential over a frequency range of 100 kHz to 10 mHz with an AC amplitude of 5 mV. The EIS data were fitted using an equivalent circuit model. The specific capacitance (*C*, F g^−1^) of the working electrode was calculated using Equation (1).

(1) $$C=\frac{I_{d}\times ∆t}{∆V\times m}$$

where *I_d_* (A) is the discharge current, Δ*t* (s) is the discharge time, Δ*V* (V) is the potential interval, and *m* (g) is the mass of active material on the working electrode.

## 3. Results

### 3.1. Chemical Structure of LRF Gels

**Figure 1:**
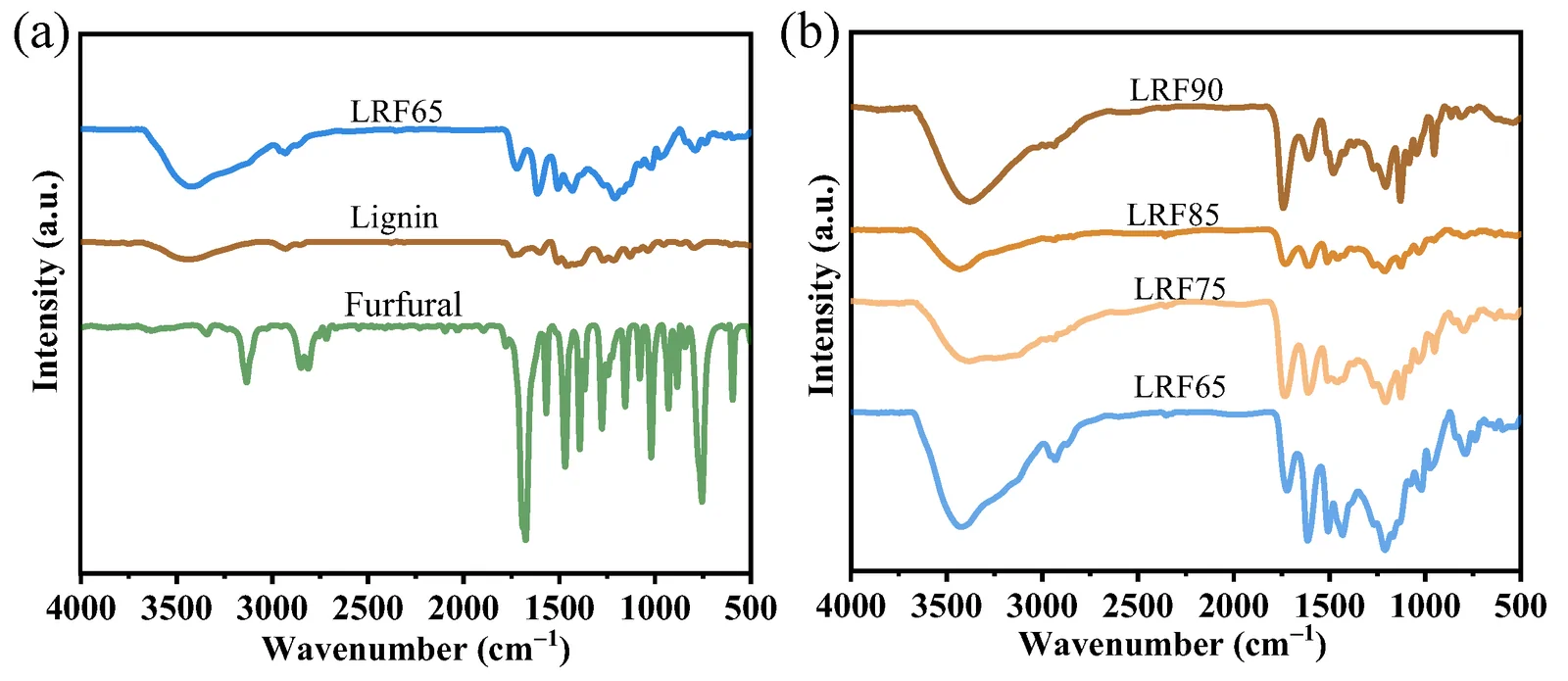
(**a**) FTIR spectra of furfural, lignin, and LRF65; (**b**) FTIR spectra of LRF samples.

Figure 1 presents the FTIR spectra of lignin, furfural, and LRF samples prepared with various lignin substitution ratios. As shown in Figure 1a, the characteristic absorption bands corresponding to the aldehyde C=O and C–H stretching vibrations of furfural (observed at 3136, 1620, and 1390 cm^−1^) are virtually absent in the LRF65 spectrum, indicating that polymerization between furfural and lignin has occurred [17]. Meanwhile, the O–H stretching band of lignin at approximately 3400 cm^−1^ remains discernible but exhibits a marked increase in intensity, suggesting that the hydroxyl groups participate in the crosslinking reaction, accompanied by the formation of hydrogen bonds and ether linkages. This inference is further supported by the enhanced C–O stretching absorption in the 1000–1200 cm^−1^ region, characteristic of crosslinked networks, particularly C–O–C ether bridges. Additionally, the aromatic ring skeletal vibrations in the 1450–1600 cm^−1^ region become broadened and coalesced, reflecting the formation of a homogeneous copolymer network via crosslinking among the benzene rings of lignin and resorcinol and the furan ring of furfural [20]. Moreover, the sharp and intense absorption features in the fingerprint region are significantly diminished, further confirming the transformation of the small-molecule precursor mixture into a polymer gel network. Notably, all LRF samples (Figure 1b) exhibit a similar broad O–H stretching band near 3430 cm^−1^ and the bands at 1610 and 1510 cm^−1^. As lignin substitution ratio increases, the C–H stretching band at 1427 cm^−1^ and the O–H bending band of phenolic moieties at 1205 cm^−1^ gradually decrease in intensity, indicating the incorporation of lignin through phenolic condensation [21]. The bands at 1120 and 950 cm^−1^ correspond to the C–O–C stretching of the furan ring and the C–H out-of-plane bending of the furan ring attached to the benzene ring, respectively. These bands signify the formation of phenyl–CHF–phenyl linkages (where F denotes the furan ring) during the crosslinking process [22].

**Figure 2:**
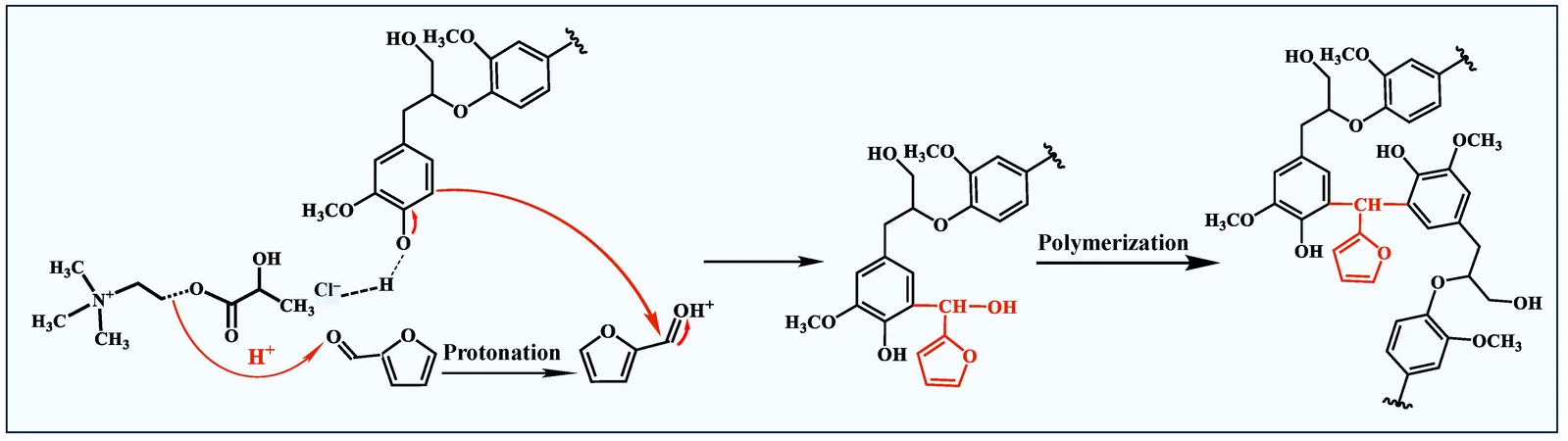
Illustration of hypothetical polymerization mechanism of furfural and lignin in DES.

The gelation time of the LRF system exhibited a pronounced dependence on the lignin substitution ratio. Samples LRF65 and LRF75 reached gelation within 3 h, whereas increasing the substitution ratio to 85% (LRF85) and 90% (LRF90) prolonged the gelation time to 4 and 5 h, respectively. Notably, all furfural-based LRF gels required considerably longer gelation periods than their formaldehyde-based counterpart (2 h) [19]. This discrepancy can be primarily attributed to the substantially higher reactivity of formaldehyde, which, as a small-molecule crosslinker, enables rapid formation of methylene bridges with phenolic compounds [23]. In contrast, the furan ring of furfural brings substantial steric hindrance during polycondensation, which may considerably retard crosslinking kinetics [24]. Beyond furan-ring steric hindrance, gelation retardation could arise from multiple coupled factors, including lignin functional-group distribution, accessibility of reactive sites, and precursor viscosity. Despite this difference, the underlying gelation mechanism of furfural with phenolic species is proposed by analogy with our previous lignin–formaldehyde under DES conditions [19]: the aldehyde group undergoes protonation by H^+^ derived from the acidic DES, generating –CHF–OH intermediates with lignin and resorcinol, which are subsequently dehydrated to form Ar–CHF–Ar linkages, ultimately culminating in the construction of a stable 3D gel network [17]. The hypothetical polymerization mechanism of furfural and lignin in DES is illustrated in Figure 2.

### 3.2. Physicochemical Properties of LRFC

**Figure 3:**
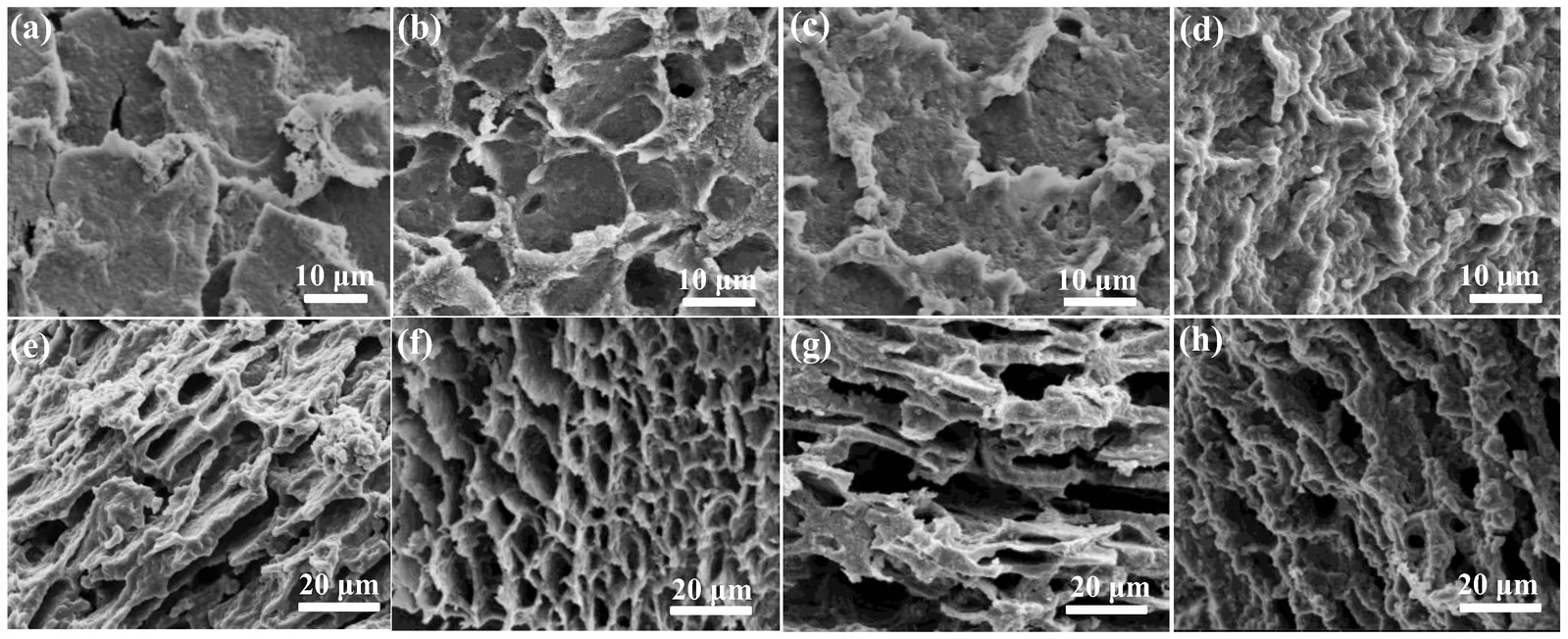
SEM images: (**a**,**e**) LRFC65; (**b**,**f**) LRFC75; (**c**,**g**) LRFC85; (**d**,**h**) LRFC90.

The morphological characteristics of LRFC samples prepared with various lignin substitution ratios were analyzed using SEM (Figure 3a–d). Increasing the lignin content induces a progressive surface morphological transformation, shifting from a lamellar architecture to agglomerated fused carbon particulates. Cross-sectional SEM micrographs (Figure 3e–h) further reveal that, although all LRFC specimens maintain a stacked porous framework, their porous architectures varied significantly with the level of substitution. Specifically, LRFC65 exhibits an interconnected layered macroporous structure with disordered interlaced wrinkled carbon sheets and abundant open macroporous channels, whereas LRFC75 developed a more regular honeycomb-shaped 3D interconnected network with thinner pore walls and more uniform macropores. These structural features reveal that an optimized lignin-to-resorcinol ratio promotes homogeneous crosslinking and yields a robust scaffold. At a higher substitution ratio, however, the porous architecture gradually deteriorated into a collapsed stripe-like morphology accompanied by reduced interconnectivity. Collectively, these findings show that the lignin substitution ratio exerts a critical influence on both the crosslinking uniformity and the overall network architecture of the resulting LRFC [18]. Furthermore, the observed morphology differs distinctly from the bead-like network assembled by carbonaceous nanospheres reported for formaldehyde-derived LRFC [19]. Accordingly, the variations in gelation behavior and final network structure arise from the combined effects of lignin precursor proportion and effective reactive groups from furfural.

**Figure 4:**
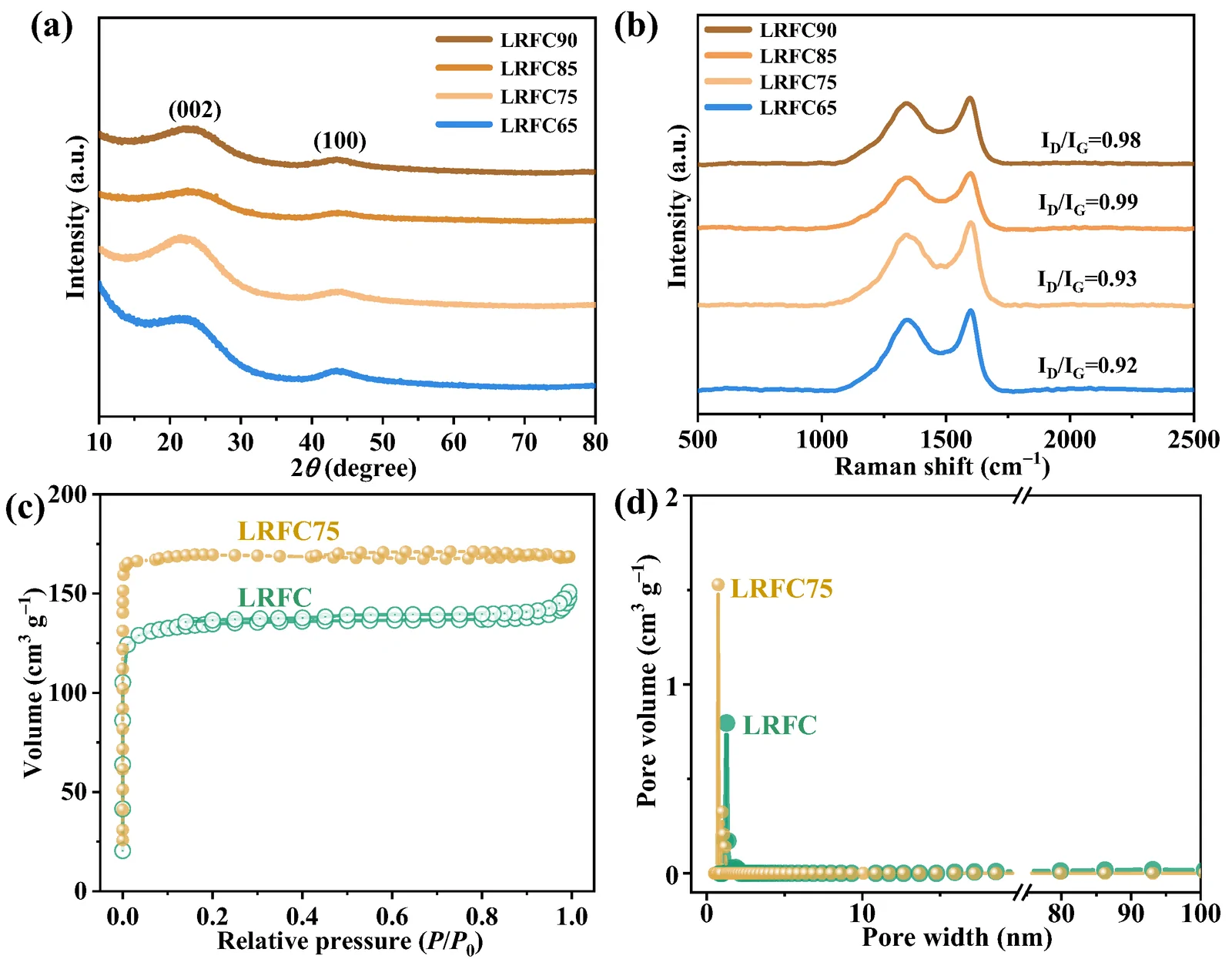
(**a**) XRD patterns and (**b**) Raman spectra of LRFC samples; (**c**) N_2_ adsorption/desorption isotherms and (**d**) pore size of LRFC75 and the reported LRFC [19].

The XRD patterns of LRFC samples with various lignin substitution ratios are presented in Figure 4a. All samples exhibit the broad diffraction peaks at approximately 23° and 43.5°, corresponding to the (002) and (100) planes, respectively, without sharp crystalline reflections detected. This pattern is characteristic of a typical amorphous carbon structure, featuring only short-range ordered stacking of carbon layers and a lack of well-defined graphite crystallites [25,26]. Notably, as the lignin substitution ratio increases, the diffraction intensity near 43.5° gradually diminishes, a trend closely associated with the proliferation of micropores within the carbon matrix [27]. This observation is further corroborated by I_D_/I_G_ ratios derived from the Raman spectra (Figure 4b). The spectra display two characteristic bands at approximately 1350 cm^−1^ (D-band, associated with structural defects) and 1580 cm^−1^ (G-band, attributed to the in-plane vibrational modes of sp^2^-hybridized aromatic rings), indicating a predominantly amorphous carbon framework with minor short-range ordering. The I_D_/I_G_ values for the as-prepared LRFC materials fall within the range of 0.9–1.0, reflecting a moderately low graphitization degree. Hence, the carbon skeleton is primarily composed of disordered amorphous carbon, interspersed with small sp^2^-hybridized graphitic domains and abundant structural defects [28]. For performance comparison, our previously reported lignin–formaldehyde-derived LRFC with a 75% lignin substitution ratio was employed [19]. Relative to this formaldehyde-based LRFC reference (I_D_/I_G_ ≈ 1.3) [19], the furfural-derived samples exhibit a distinctly lower I_D_/I_G_ ratio. This decline originates from fewer oxygen-containing heteroatoms introduced into the polymer network during furfural polymerization, which in turn decreases the number of structural defect sites [29]. Despite obvious discrepancies in surface morphology, both furfural-derived and formaldehyde-based LRFC samples display type I isotherms at the same 75% lignin substitution ratio (Figure 4c), which is characteristic of microporous materials. The LRFC75 sample exhibits a specific surface area of 471 m^2^ g^−1^ and an average pore width of 1.41 nm (Figure 4d). Significantly, the microporous structure with pore sizes around 1 nm can provide abundant electroactive sites and favorable pore dimensions for electrolyte ion adsorption [3,30].

### 3.3. Electrochemical Performance of LRFC

**Figure 5:**
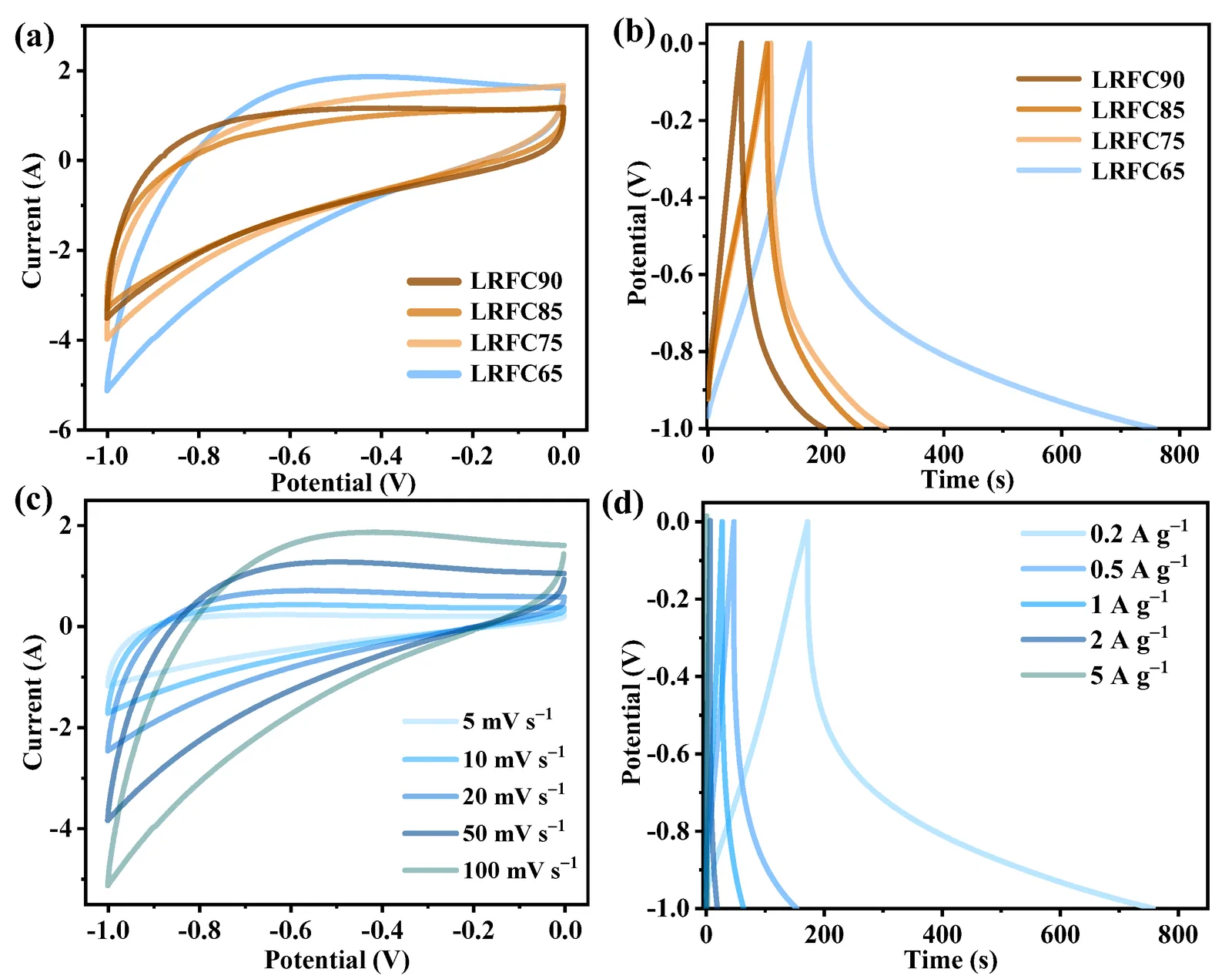
(**a**) CV curves at 100 mV s^−1^ and (**b**) GCD curves at 0.2 A g^−1^; (**c**) CV curves at different scan rates and (**d**) GCD curves at various current densities of LRFC65 electrode.

To evaluate the electrochemical performance of LRFC samples prepared with various lignin substitution ratios, the CV, GCD, and EIS measurements were conducted in a three-electrode system using a 6 M KOH electrolyte. As shown in Figure 5a, all LRFC electrodes exhibit quasi-rectangular CV curves without distinct redox peaks, which is characteristic of typical electric double-layer capacitance (EDLC). Notably, the CV curves of the LRFC65 electrode are well retained in a nearly rectangular shape even at elevated scan rates from 5 to 100 mV s^−1^ (Figure 5c), confirming rapid ion/electron transfer kinetics and excellent electrochemical reversibility [14,26]. Among the samples, LRFC65 delivers the largest integrated CV area at 100 mV s^−1^, reflecting the highest gravimetric specific capacitance, followed by LRFC75. In contrast, LRFC85 and LRFC90 show a substantial decline in capacitive performance, which can be attributed to the excessive lignin content that induces pore structure densification and disordered crosslinking within the carbon framework. Therefore, these results demonstrate that the lignin substitution ratio not only influences the porous architecture and structural ordering of the carbon framework but also serves as a critical parameter for tuning electrochemical performance. Notably, a moderate lignin substitution ratio of 65% is found to be optimal for achieving superior capacitance performance.

The GCD curves of LRFC samples with various lignin substitution ratios at a current density of 0.2 A g^−1^ are shown in Figure 5b. All samples exhibit near-triangular profiles with noticeable deviations from ideal symmetry, indicating non-ideal capacitive behavior. Specifically, the LRFC65 electrode maintains its characteristic asymmetric near-triangular shape in current densities of 0.2–5 A g^−1^ (Figure 5d). This asymmetry is primarily attributed to the heterogeneous pore structure, which results in ion diffusion kinetics across intrinsic micropores and slit-like pores from stacked sheets [3]. In particular, ions tend to preferentially migrate into macropores during charging, while their release from micropores and tight stacking slit-like pores are kinetically hindered during discharging, leading to an asymmetric temporal distribution in the charge–discharge response [2]. Among all the tested samples, LRFC65 delivers the highest specific capacitance of 117.2 F g^−1^ at 0.2 A g^−1^. The other calculated specific capacitances are 39.3, 31.9, and 28.2 F g^−1^ for LRFC75, LRFC85, and LRFC90, respectively. The exceptional capacitive behavior of LRFC65 is attributed to its interconnected layered macroporous structure with disordered interlaced wrinkled carbon sheets combined with decent graphitic conductivity, which offer abundant ion-accessible sites for charge storage and yield high specific capacitance. In addition, moderate oxygen-containing functional groups within LRFC materials can enhance electrolyte wettability and accelerate ion transport into internal pores, thereby introducing considerable pseudocapacitive contribution beyond EDLC [19,31].

Moreover, Table 1 summarizes the preparation methods and key properties of CAs derived from various precursors. The comparison reveals that formaldehyde exhibits higher crosslinking reactivity than bio-based furfural, which contributes to improved specific capacitance in lignin–formaldehyde systems. In contrast, furfural serves as a non-toxic and fully renewable alternative. When polymerized with lignin, furfural tends to construct a sheet-like network architecture, which is distinctly different from the bead-like chain morphology typically observed in lignin–formaldehyde systems [31]. Additionally, lignin subunits and synthetic methods exert significant impacts on the final performance of the as-prepared CAs. Template-assisted strategies (e.g., salt-template and ice-template) can effectively enhance the BET surface area [32,33]. Nevertheless, a large surface area alone cannot guarantee high capacitance, since pore connectivity and carbon conductivity jointly determine the overall electrochemical behavior. In the DES system, lignin–furfural CA synthesized by the sol–gel method achieves a competitive surface area, and further activation or template modification is expected to enhance its capacitive performance.

Against this background, the as-prepared LRFC in this work presents clear advantages, that is, lignin and furfural are abundant, renewable, and environmentally benign precursors, enabling a sustainable fabrication route, and the present synthetic strategy is simple, low-cost, and time-efficient. It achieves a favorable trade-off between renewable feedstocks, simple processing, low manufacturing cost, and satisfactory electrochemical performance. These combined merits make LRFC materials promising for sustainable electrode applications when compared with mainstream graphene-derived electrodes and conventional fossil-based porous carbons. It should be noted that the electrochemical performance evaluation of as-prepared electrodes in this work is limited to a three-electrode test configuration. Long-term cycling stability tests and symmetric two-electrode cell measurements were not performed in the current study, which will be further investigated in our follow-up research.

**Figure 6:**
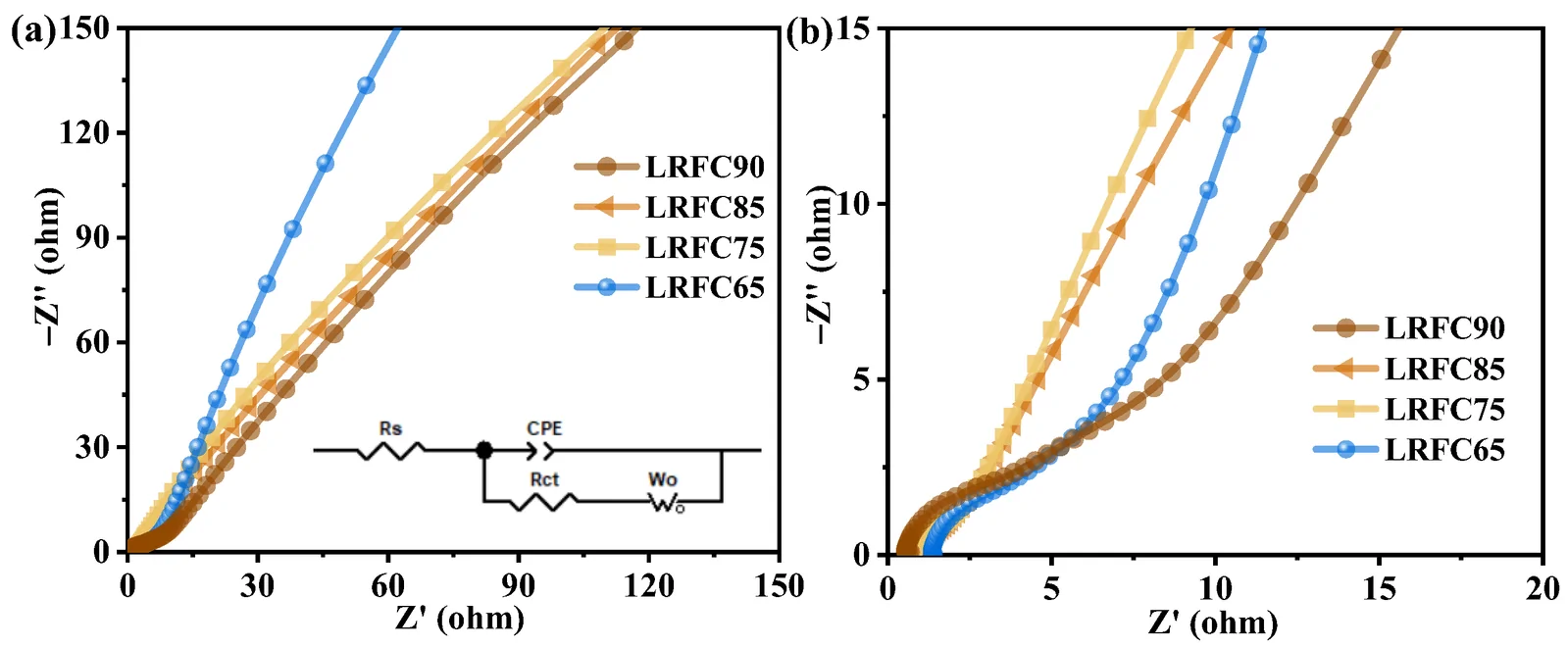
(**a**) EIS spectra with inserted equivalent circuits and (**b**) high-frequency EIS profiles of LRFC samples with various lignin substitution ratios.

The EIS spectra are further analyzed to evaluate the impedance behavior of LRFC electrodes fabricated with various lignin substitution ratios. The Nyquist plots are fitted using the equivalent circuit *R*_s_(*R*_ct_*W*_o_)//CPE (inset in Figure 6), and the resulting impedance parameters, along with the chi-squared (χ^2^) goodness-of-fit values, are summarized in Table 2. All fitted EIS spectra yield χ^2^ values below 0.01, and relative errors (Error%) of each circuit parameter fall below 5, within the well-accepted credible range for EIS fitting. These low fitting errors indicate that the adopted equivalent circuit closely reproduces experimental impedance data. No significant fitting deviation or overfitting is observed, which supported the credibility of this impedance analysis and the derived electrode structural features. The extracted equivalent series resistance (*R*_s_), charge transfer resistance (*R*_ct_), and constant-phase element (CPE) parameters are rational.

Furthermore, the CPE-P exponent (0 < *P* ≤ 1) is commonly encountered for porous-carbon electrodes during EIS measurements. Deviations of P from unity arise from surface inhomogeneity, porous effects, and distributed relaxation times at the electrode/electrolyte interface. All samples yield CPE-P values above 0.9, indicative of near-ideal capacitive behavior. Among the as-prepared electrodes, the larger CPE-T value of LRFC65 suggests a lower interfacial impedance and improved charge/storage capability. Meanwhile, LRFC65 exhibits the steepest slope in the low-frequency region, indicating the lowest ion diffusion resistance (*W*_o_) within its interconnected macroporous network [34,35]. The *R*_s_ values of all the LRFC electrodes range from 0.5 to 1.3 Ω, indicating favorable current collector contact and adequate electrolyte wettability [14]. Notably, LRFC75 displays a smaller *R*_ct_ value (1.095 Ω), suggesting accelerated charge transfer kinetics originating from its highly ordered architecture and good pore interconnectivity. Nevertheless, a low *R*_ct_ primarily reflects favorable electronic conductivity and rapid interfacial charge transfer, yet it cannot solely govern the final capacitance performance. By contrast, LRFC65 preserves its interconnected layered macroporous structure with disordered interlaced wrinkled carbon sheets together with decent graphitic conductivity, providing abundant ion-accessible sites for charge storage, which dominates its higher specific capacitance even with a relatively larger *R*_ct_ (3.316 Ω). Overall, variations in these impedance parameters closely correlate with differences in defect density, graphitization degree, and pore architecture induced by varying lignin substitution.

## 4. Conclusions

In summary, this study elucidates the pivotal role of lignin substitution ratio in determining the gelation behavior, microstructure, and electrochemical performance of LRFC materials synthesized in an efficient DES-based system. Increasing the lignin substitution ratio from 65% to 90% prolongs gelation time and induces a nonstructural transition from interconnected fibrous networks to dense lamellar structures with poor pore interconnectivity. The LRFC65 electrode achieves the optimal specific capacitance of 117.2 F g^−1^ at 0.2 A g^−1^, alongside lower charge transfer and diffusion resistances. This work presents a novel, sustainable route integrating lignin, bio-based furfural, and DES for high-performance CA electrodes, establishing the lignin substitution ratio as a simple yet effective parameter for performance tuning. Beyond mechanistic insights, this study offers a promising strategy for lignin and furfural valorizations and the development of sustainable energy storage biomaterials.

## Figures and Tables

**Table 1:** Comparison of key properties of CAs derived from various precursors.

Samples	Precursors	Method	Surface Area (m^2^ g^−1^)	Specific Capacitance (F g^−1^)	Refs.
Wheat straw LRFC	Lignin + formaldehyde	Sol–gel	421	284 (0.5 A g^−1^)	[19]
Poplar LRFC	Lignin + formaldehyde	Sol–gel	64.9	88.1 (0.5 A g^−1^)	[31]
Pine LRFC	Lignin + formaldehyde	Sol–gel	5.89	197 (0.5 A g^−1^)	[31]
Wheat straw LRFC	Lignin + formaldehyde	Sol–gel	159.1	98.7 (0.5 A g^−1^)	[31]
LTCA	Lignin + TOCNF	Ice-template	806	124 (0.2 A g^−1^)	[32]
RFCA	Resorcinol + furfural	Salt-template	548	—	[33]
Pine LRFC	Lignin + furfural	Sol–gel	471	117 (0.2 A g^−1^)	This work

**Table 2:** All fitted impedance parameters of LRFC electrodes.

Sample	*R*_s_ (Ω)	*R*_ct_ (Ω)	CPE-T (F s^P−1^)	CPE-P	$\chi^{2}$	Error%
LRFC65	1.304	3.316	6.2 × 10^−4^	0.912	9.6 × 10^−3^	2.453
LRFC75	0.735	1.095	1.2 × 10^−4^	0.955	8.0 × 10^−3^	4.645
LRFC85	1.036	1.902	2.2 × 10^−4^	0.914	4.2 × 10^−3^	5.004
LRFC90	0.536	5.487	2.7 × 10^−4^	0.921	4.3 × 10^−3^	1.437

## Data Availability

The original contributions presented in this study are included in the article, further inquiries can be directed to the corresponding author.
